# First person – Yuta Koui

**DOI:** 10.1242/bio.059608

**Published:** 2022-09-15

## Abstract

First Person is a series of interviews with the first authors of a selection of papers published in Biology Open, helping researchers promote themselves alongside their papers. Yuta Koui is first author on ‘ Hepatic leukemia factor-expressing paraxial mesoderm cells contribute to the developing brain vasculature’, published in BiO. Yuta is a postdoctoral fellow in the lab of Yoh-suke Mukouyama at the Laboratory of Stem Cell and Neuro-Vascular Biology, Cell and Developmental Biology Center, National Heart, Lung, and Blood Institute, National Institutes of Health, Bethesda, USA, investigating cellular origin of brain vascular cells developing CNS-specific unique vascular structure such as blood brain barrier.


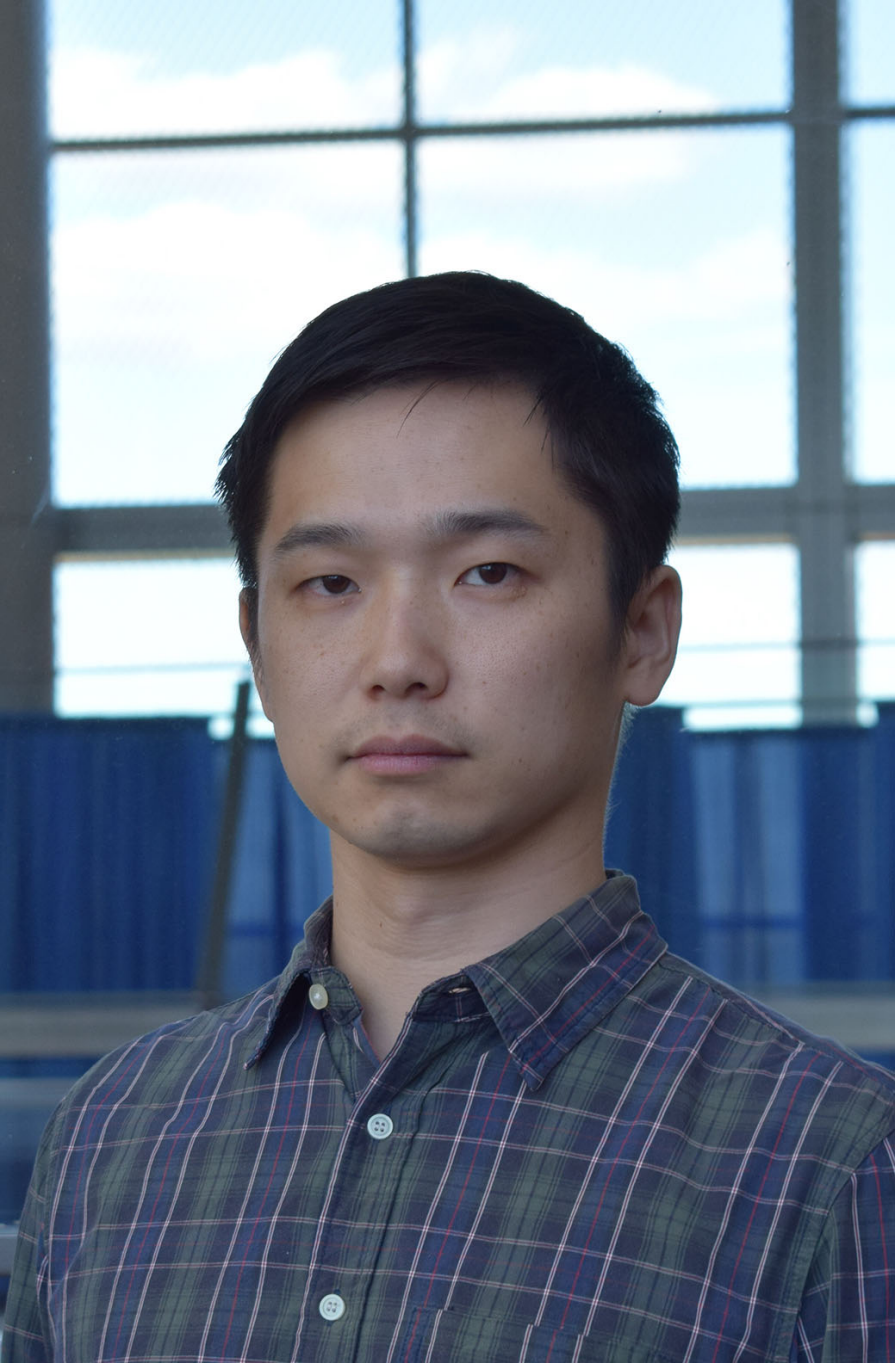


**Yuta Koui**

**Describe your scientific journey and your current research focus**

My research interest is studying how various types of cells communicate with each other to develop organ-specific functional properties. My PhD work focused on the crosstalk between hepatocytes and non-parenchymal cells such as liver sinusoidal endothelial cells and hepatic stellate cells in liver development and liver diseases. As a postdoctoral fellow, I am studying functional interactions between nervous and vascular systems: my interdisciplinary research program focuses on two major neuro-vascular interfaces. The first neuro-vascular interface is the coordination and integration of functional branching patterns of sensory nerves and blood vessels in the skin. The second neuro-vascular interface is a brain-specific unique vascular structure that establishes blood brain barrier (BBB) formation. As a first step towards characterizing brain vascular cells in the latter project, I decided to characterize the cellular origin of brain endothelial cells and accessory pericytes. Our lineage tracing studies clearly demonstrate that hepatic leukemia factor (Hlf)-expressing cephalic paraxial mesodermal cells contribute to endothelial cells and pericytes in the developing brain vasculature.

**How would you explain the main finding of your paper?**

Blood vessels compose the indispensable circulatory network for every tissue to transport oxygenated or deoxygenated blood and maintain organ-specific function by interacting with tissue parenchymal cells. Blood vessels in the brain are highly specialized because they develop the BBB, which limits transportation of substances between the bloodstream and the brain parenchyma. These unique properties raise a question of how endothelial cells and accessory pericytes of blood vessels emerge during brain development. We identified a unique expression of hepatic leukemia factor (*Hlf*) in the head mesenchyme of mouse embryos and discovered that Hlf marks head mesenchymal cells, which mainly contribute to endothelial cells and pericytes of blood vessels in the brain. Taken together, our findings suggest that Hlf-expressing head mesenchymal cells generate brain endothelial cells and pericytes.

**What are the potential implications of this finding for your field of research?**

Historically, mesoderm differentiates into endothelial cells and pericytes, but these brain vascular cells derived via a classical pathway of mesodermal differentiation into endothelial cells and pericytes remain to be investigated. Our lineage tracing studies provide evidence that Hlf-expressing cephalic mesenchymal cells contribute to endothelial cells and pericytes in the developing brain vasculature.

**Figure:**
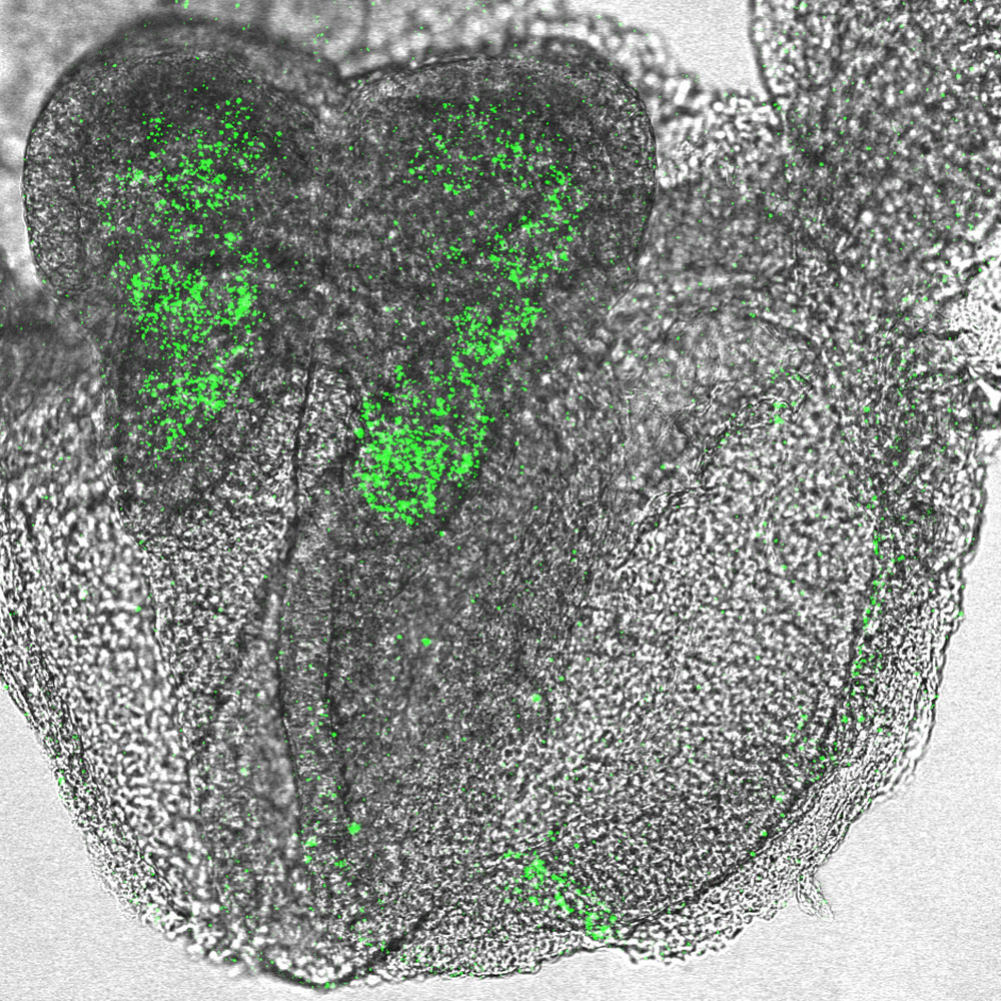
**A unique expression of hepatic leukemia factor (*Hlf*) in the cephalic paraxial mesenchyme: RNA whole-mount *in situ* hybridization chain reaction of E8.5 wild-type embryos revealed the expression of *Hlf* (green) in the cephalic region.** We showed that Hlf-expressing cephalic paraxial mesenchymal cells contribute to the developing brain vascular cells such as endothelial cells and pericytes.

**Which part of this research project was the most rewarding?**

This is a project that only became possible when two dynamic collaborations occurred: first, Dr Yokomizo's team at IRCMS, Kumamoto University, developed lineage-tracing tools to mark Hlf-expressing cephalic mesenchymal cells; second, my NCI colleagues (Drs Anderson and Boylan) helped to establish high resolution whole-mount RNA *in situ* hybridization chain reaction.

**What do you enjoy most about being an early-career researcher?**

Working as an early-career researcher gives me a great opportunity to develop my scientific career. In my current laboratory at the National Heart, Lung, and Blood Institute, I enjoy interdisciplinary collaborations that allow me to view my current projects and experimental approaches from a new perspective.

**What's next for you?**

Given that our lineage tracing studies indicate the contribution of Hlf-expressing cephalic mesenchymal cells in the vasculature of hindbrain, midbrain, and diencephalon but rarely in the cerebral cortex, it is interesting to examine what controls such differences in the contributions of Hlf-expressing cells. From the aspect of my research career, my broad goal is to make a lasting contribution to the scientific community, not only with primary research findings, but also by providing mentorship to the next generation of junior scientists.

## References

[BIO059608C1] Koui, Y., Ideue, T., Boylan, M., Anderson, M. J., Osato, M., Suda, T., Yokomizo, T. and Mukouyama, Y.-s. (2022). Hepatic leukemia factor-expressing paraxial mesoderm cells contribute to the developing brain vasculature. *Bio. Open* 11, bio059510. 10.1242/bio.059510 36017733 PMC9493726

